# Cognitive fusion and personality traits in the context of mindfulness: A cross-sectional study

**DOI:** 10.1371/journal.pone.0273331

**Published:** 2022-09-28

**Authors:** Sarah Pux, Eric Hahn, Niklas Bergmann, Inge Hahne, Luca Pauly, Thi Minh Tam Ta, Gerdina H. M. Pijenborg, Kerem Böge

**Affiliations:** 1 Department of Psychiatry and Psychotherapy, Charité–Universitätsmedizin Berlin, Berlin, Germany; 2 Department of Clinical and Developmental Neuropsychology, University of Groningen, Groningen, The Netherlands; 3 Department of Psychotic Disorders, Assen, The Netherlands; University of St Andrews, UNITED KINGDOM

## Abstract

**Objectives:**

Meditation and mindfulness, though rooted in eastern traditions, have been increasingly embraced in western psychology. However, questions remain regarding the mechanisms through which the beneficial effects of mindfulness occur. The present study aimed to address cognitive fusion as a potential mediator between mindfulness and negative affect and whether the mindfulness-cognitive fusion link is moderated by personality factors in an international sample of frequent meditators.

**Methods:**

An international sample of 739 frequent meditators completed measures of mindfulness (Southampton Mindfulness Questionnaire), cognitive fusion (Cognitive Fusion Questionnaire), negative affect (Brief Symptom Checklist), and personality (Big Five Inventory) in an online survey. Using SPSS Process Macro, cognitive fusion was examined as a mediator between mindfulness and negative affect. Furthermore, Extraversion, Conscientiousness, and Neuroticism were investigated as moderators in the mediation model.

**Results:**

Cognitive fusion was found to be a partial mediator between mindfulness and negative affect (*b* = -0.249; 95% *CI*, [-0.289, -0.203]), whereas the examined personality factors were not found to moderate the present model (E: *t*(734) = 0.951, *p =* .342); C: *t*(734) = -0.042, *p* = .967; N: *t*(734) = -2.271, *p* = .023).

**Conclusions:**

This study suggests that cognitive fusion plays a significant role in the association between mindfulness and negative affect. These findings indicate the importance of how individuals internally respond and relate to experiences and the instrumental value of mindfulness effects beyond and outside of mindfulness-based interventions.

## Introduction

Meditation, an umbrella term encompassing a wide range of practices with roots in ancient, Eastern contemplative traditions, has in recent decades found increasing recognition in academic psychology. Particularly since the introduction of mindfulness to Western psychology by Kabat-Zinn, mindfulness and meditation have been researched abundantly and implemented in various approaches of psychotherapy, including Acceptance Commitment Therapy [1]. Conceptualized as a way of paying attention with a focus on awareness, the present moment and non-judgment [2, 3], the construct of mindfulness can be considered a heightened form of attention, and as such, a cognitive phenomenon itself [4, 5]. As a corollary of this operationalization, mindfulness can be viewed as a psychological measure of intrinsic capacities that can be cultivated through meditation [6].

On process-based levels, positive effects of mindfulness have been shown for well-being [7], negative affect [8], emotion regulation [9], and cognitive flexibility [10], demonstrated in both clinical and nonclinical populations [11–14]. Particularly in populations of experienced meditators, mindfulness and meditation experience are positively associated with attention regulation, body awareness and emotion regulation [6, 15]. However, amid ample research in the field, scholars have called for further investigation into how meditation exerts its benefits, as well as individual differences which may be boundary conditions of meditation effects [16, 17].

Among the various proposed mechanisms underlying the effects of mindfulness is the concept of cognitive fusion [18], which is defined as a tendency of behavior to be overly regulated by internal experiences [19]. Cognitive fusion is a central part of the broader term psychological flexibility, conceptualized as an openness and acceptance of present experiences, allowing flexibility to adapt to one’s environment [18]. Cognitive fusion has frequently been associated with poorer mental health outcomes, such as depression and anxiety [20] and has been proposed to be one of the core processes that may explain the link between vulnerabilities and psychopathology [21]. Moreover, cognitive fusion has continually been related to negative affect, as affirmed in evidence of ACT treatment effects [22], to rigidity as a feature of affective disorders [23] and to maladaptive emotion regulation strategies [24, 25]. Notably, cognitive fusion intrinsically represents a contrast to mindfulness skills (i.e., nonreactivity and nonjudging), which have been shown to be negatively associated with negative affect [26]. Similarly, the negative association between mindfulness and negative affect, conceptualized as a person’s experience of unpleasant arousal and distress [27, 28], has consistently been reported [1, 13, 29, 30]. Tying the evidence together [31], reported results of cognitive fusion as a mediator in the relationship between dispositional mindfulness and negative affect in children.

All three constructs addressed, namely (1) mindfulness, (2) cognitive fusion as part of psychological flexibility, and (3) negative affect have been shown to be associated with personality, specifically with neuroticism, conscientiousness and extraversion [32, 33]. Personality traits refer to relatively stable and enduring individual differences in patterns of thoughts, feelings, and behaviors, influencing how we interact with the world. For example, individuals with high scores on neuroticism have been shown to be less psychologically flexible while individuals with high extroversion have been shown to be more psychologically flexible [34]. Given the evidence of such associations between personality factors and psychological flexibility, it is of importance to investigate whether the relationship between an adaptive skill like mindfulness and psychological flexibility, which is thought to be increased by mindfulness, is impacted by presence of specific personality traits. Despite limited research on the role of personality characteristics in mindfulness, personality has previously been proposed to play a moderating role in mindfulness, based on findings of greater effects of mindfulness training in students with the personality traits of neuroticism and conscientiousness by [35]. While mindfulness and psychological flexibility have repeatedly been shown to be associated with personality, so far only one study has investigated the interrelation of all three constructs. According to [32], neuroticism was negatively associated with mindfulness and psychological flexibility while conscientiousness was positively associated with mindfulness and psychological flexibility. Extraversion was reported to be positively associated with psychological flexibility [33], however both negative and positive correlations between extraversion and mindfulness were found in the literature [36].

Given the significant associations between mindfulness, cognitive fusion, negative affect, and personality, the present study proposes a moderated atemporal mediation model to elucidate the mechanisms underlying the benefits of mindfulness for negative affect in a cross-sectional design. Based on the substantial role these variables play, individually and in combination, with regard to the risk of negative affect and distress it is important to examine their effect in a combined model. To our knowledge, this study is the first to investigate the above constructs in one model and specifically in a population of meditators from the general population. As amount of time spent on meditation practice was shown to be associated with mindfulness scores on self-report questionnaires [37], it can be reasoned that the effect between the above-mentioned variables will be particularly present in this diverse sample of meditators including a wide range of levels of mindfulness experience. Therefore, investigating the effect in this population elucidates effects of frequent mindfulness practice on negative affect outside of clinical application, but with an implementation of these practices comparable to clinical interventions.

With this framework, we aim to contribute to a theory driven model of the benefits of mindfulness, which is currently lacking. The relevance of this investigation is two-fold. For one, insight into the defining mechanisms through which state mindfulness is associated with beneficial effects, is an important addition to the literature of evidence-based mindfulness-based treatment, contributing to the field of clinical application Secondly, identifying mediating factors in the effects of mindfulness, as well as the role personality characteristics play in this relationship may suggest important factors in the development and maintenance of adaptive coping and emotion regulation strategies in a population of meditators.

The present study aims to address the question whether the association between mindfulness and negative affect can be explained by cognitive fusion. Further, the question whether this mediation model is moderated by personality characteristics will be examined. To investigate the proposed model, four hypotheses will be tested: It is hypothesized that (1) levels of mindfulness are negatively associated with negative affect; (2) levels of cognitive fusion are positively associated with negative affect; (3) cognitive fusion partially mediates the relationship between mindfulness and negative affect and lastly, (4) the personality traits of neuroticism, conscientiousness, and extraversion moderate the relationship between mindfulness and cognitive fusion.

## Method

### Design

An international cross-sectional online survey-based design was used to assess meditators from the general populations’ levels of cognitive fusion, and its mediating role in the relationship between mindfulness (IV) and negative affect (DV). Further, the personality factors of extraversion, conscientiousness, and neuroticism were examined as moderators of the relationship between mindfulness and cognitive fusion. Due to the cross-sectional nature of this design, this study assesses an atemporal meditation model, as recommended by Winer et al. [38].

### Participants

Participants were 739 adults, recruited from a population of frequent meditators between June 2020 and August 2021. The sample consisted to 70.5% of females, 28.2% males, 1.3% diverse (referring to gender), with a mean age of 41 (*SD* = 16.5) years. Participants were from Europe (51.3%), North America (31.1%), Australia and New Zealand (9.3%), Asia (6.6%), South America (0.9%), Africa (0.8%). On average, participants meditated 4.76 (*SD* = 9.25) hours per week and had 6.79 (*SD* = 10.5) years meditation experience. Participation in the study was not compensated. Inclusion criteria were specified as being at least 18 years of age and proficiency in the English language. In addition, to be included as a “meditator” a minimum meditation practice of 1 month, also applied in previous studies in the literature [39–41], as well as weekly meditation was specified to assure sufficient practice to be able to retain the mindfulness skills this study set out to measure while allowing a diverse range of meditators to be included The cut-off was intended to include a minimum amount of experience that would be comparable to mindfulness-based clinical interventions (e.g.: MBSR) while still allowing for a diverse range of meditators to be included in the sample. Other studies using this particular cut-off include [39–41]. In total, 950 people completed the questionnaire, of which 161 responses were excluded. Participants were excluded based on insufficient meditation experience and incompatible responses on demographic questions (i.e., age incompatible with meditation experience in years, extremely high age).

### Procedure

Ethical approval was granted by the Charité–Universitätsmedizin Berlin ethics committee (Reference: EA4/127/20). Recruitment was carried out via meditation oriented online platforms including meditation teacher registries as well as secular and non-secular meditation communities and organizations posting a brief explanation and invitation to the study. The study was performed from June 2020 to August 2021 in form of an online questionnaire via the online survey platform Unipark Software Questback. After giving written informed consent, participants were presented with demographic questions, inquiries about meditation practice and history of psychopathology. Subsequently, five English-language questionnaires were presented. Completion of the study took approximately 20 minutes.

### Materials

The present study examined four psychological constructs, namely mindfulness, cognitive fusion, negative affect, and personality with the following self-report questionnaires.

### Mindfulness

The Southampton Mindfulness Questionnaire (SMQ; [42]) consists of 16 items, scored on a 7-point Likert scale (0 = strongly disagree, 6 = strongly agree). The SMQ addresses state mindfulness, in particular the degree of mindful responding to distressing cognitions (thoughts, images, and perceptions) and has good psychometric properties (α = .89; [42, 43]). In the present sample, Cronbach’s α was .92.

### Cognitive fusion

Cognitive fusion was assessed with the Cognitive Fusion Questionnaire (CFQ; [20], consisting of seven items, each rated on a 7-point Likert scale (1 = never true, 7 = always true). Higher scores reflect higher fusion with inner experiences. Psychometric properties of the CFQ have been shown to be robust [44] with high internal reliability (α = .90) [20]. The Cronbach’s α in this sample was .95.

### Negative affect

The Brief Symptom Checklist (BSCL; [45]) is a short version of the Symptom Checklist-90, based on the Brief Symptom Inventory [46]. This well-validated version consists of 53 items and assesses subjective physiological and psychological symptoms including scales for fear and depression [47]. Items are rated on a 5-point Likert scale according to how much the described symptom has been experienced in the last 7 days (0 = not at all, 4 = overwhelmingly). For the present study, scores are computed by calculating a Global Severity Index (GSI), a subscale of the BSCL, with higher values representing higher symptom impact. The GSI was chosen as it combines a broad range of forms of negative affect including, but not limited to, depression and anxiety. For interpretation purposes, T-values of GSI scores were used, as recommended by [47]. In comparison to other specific measures for negative affect, like the Positive and Negative Affect Schedule (PANAS; [48]), the BSCL has been generally found to be appropriate for use in a wide variety of cultural context. In addition, while the PANAS is also widely used in the literature, has been criticized as measuring negative affect less accurately than measures that focus on discrete emotions, like the BSCL [49]. Psychometric properties were found to be good, with excellent internal validity (α = .93) for the GSI [45] and a Cronbach’s α of 0.97 in the present sample.

### Personality

In order to assess personality factors, specifically extraversion, conscientiousness and neuroticism, the Big Five Inventory [50] was used. The well validated inventory [51] consists of 44 items, rated on a 5-point Likert scale (1 = disagree strongly, 5 = agree strongly), measuring the five subdomains of the Five Factor Personality Model (extraversion, conscientiousness, neuroticism, agreeableness, and openness). High internal consistency values (α = .83) have been shown for BFI-items across samples [52]. The Cronbach’s α in this sample was 0.74 overall, and 0.840 for the Scale Extraversion, 0.759 for Agreeableness, .811 for Conscientiousness, .860 for Neuroticism and .761 for Openness. In the present study, the scores on the subscales Extraversion, Conscientiousness and Neuroticism were used.

### Statistical analysis

Data analysis was conducted using IBM SPSS 27. Sample size calculation was conducted using the simulation method in Fritz & MacKinnon [53]. The minimum sample size required for detection of a small effect on path a and b with 0.8 power using a PROCESS mediation analysis with percentage bootstrapping was estimated to be 558 participants. In order to examine the proposed moderated mediation model, the analysis was performed using the PROCESS macro [54]. To assess linearity of residuals, visual inspection of plots was carried out. Further, correlation analyses were carried out between mindfulness and negative affect, and between the mediator cognitive fusion and negative affect. Using visual inspection of plots, the normality and homoscedasticity assumptions were assessed. To address violations of homoscedasticity and normality assumptions, heteroscedasticity robust Standard Errors (HC4) and bootstrapping with 5000 estimates were applied in the analysis. Further, violations of the multicollinearity assumption concerning the moderator variables were noted but deemed not problematic, based on [54]. Subsequently, analysis of the proposed mediation model was carried out using PROCESS Model 4 to assess the mediation effect and Model 7 to examine the complete moderated meditation model with the independent variable mindfulness, the dependent variable negative affect, cognitive fusion as the mediator, and personality (extraversion, conscientiousness, and neuroticism) as the moderator and age as covariate. Reported effects refer to unstandardized effect. Further, significance was set at α = .05.

## Results

Table 1 shows sociodemographic and meditation-related characteristics of the sample. Descriptive statistics of questionnaire scores are presented in Table 2. Additional information on the sample as well as meditation types can be found in supplemental analyses. In order to establish whether the predictors were suitable for the proposed model, correlations between mindfulness and negative affect as well as between the mediator cognitive fusion and negative affect were examined, addressing the first two hypotheses (see Table 3). As predicted, mindfulness was negatively correlated with negative affect (*r* = -.589, *p <* .001) and cognitive fusion was positively correlated with negative affect (*r* = .707, *p* < .001). Based on the moderate correlations between age and the predictors, age was included as a covariate in the analysis.

**Table 1:** Sociodemographic and meditation-related characteristics.

Variables	Summary statistic–n (%)^a^	
Age (years)–mean (SD)		41.04 (16.5)
Gender		
	Female	70.5%
	Male	28.2%
	Diverse	1.3%
Belief		
	Religious	335 (42.5%)
	Agnostic	56 (7.1%)
	Atheist	86 (10.9%)
	Other	125 (15.8%)
	Nothing in particular	193 (23.7%)
Region of Residency		
	Europe	405 (51.3%)
	North America	245 (31.5%)
	Australia / New Zealand / Pacific	73 (9.3%)
	Asia	53 (6.6%)
	South America	7 (0.9%)
	Africa	6 (0.8%)
Degree of urbanity of residence		
	Megacity	36 (4.6%)
	Large metropolitan area (1.5 mio.– 10 mio. people)	161 (20.4%)
	Metropolitan area (500 000–1.5 mio. people)	139 (17.6%)
	Medium urban area (100 000 to 500 000 people)	187 (23.7%)
	Small urban area (10 000–100 000 people)	160 (20.3%)
	Rural area (< 10 000 people)	106 (13.4%)
Meditation practice variables		
	Meditation experience in years–mean (SD)	6.79 (10.05)
	Meditation intensity (hours per week)–mean (SD)	4.76 (9.25)
	Meditation experience in years–Range	0.83–60
	Meditation intensity (hours per week)—Range	0.1–130
Meditation types^b^		
	Attentional	735 (93.2%)
	Constructive	444 (56.3%)
	Deconstructive	359 (50.1%)

N = 739.

^a^ Statistics in this column are n (%) unless otherwise specified.

^b^ Participants could choose multiple options; the total percentage may exceed 100%.

**Table 2:** Descriptive statistics of questionnaire scores.

Variables	Min—Max	M (SD)
SMQ	8–96	58.05 (17.05)
CFQ	7–49	24.10 (9.41)
BSCL	39.42–101.10	50 (10)
BFI_E	1–5	3.20 (0.83)
BFI_C	1–5	3.75 (0.71)
BFI_N	1–5	2.77 (0.89)

*N* = 739, SMQ: Southampton Mindfulness Questionnaire; CFQ: Cognitive Fusion Questionnaire; BSCL: Brief Symptom Checklist; BFI: Big Five Inventory. E: Extraversion; C: Conscientiousness; N: Neuroticism.

**Table 3:** Correlations between predictors.

Variables	1	2	3	4	5	6	7	8
1 Age	-							
2 Gender	.034	-						
3 SMQ	.376**	.091**	-					
4 CFQ	-.376**	-.032	-.753**	-				
5 BSCL	-.344**	.027	-.589**	.707**	-			
6 BFI_E	.085*	-.083*	.224**	-.281**	-.310**	-		
7 BFI_C	.253**	-.157**	.318**	-.347**	-.387**	.259**	-	
8 BFI_N	-.314**	-.090**	-.650**	.738**	.633**	-.350**	-.368**	-

*N* = 739

* *p* < .05

** *p* < .01. 3 Mindfulness as measured by SMQ total score; 4 Cognitive fusion as measured by CFQ total score; 5 Negative affect in BSCL T-Scores; 6 Extraversion; 7 Conscientiousness; 8 Neuroticism.

**Fig 1:**
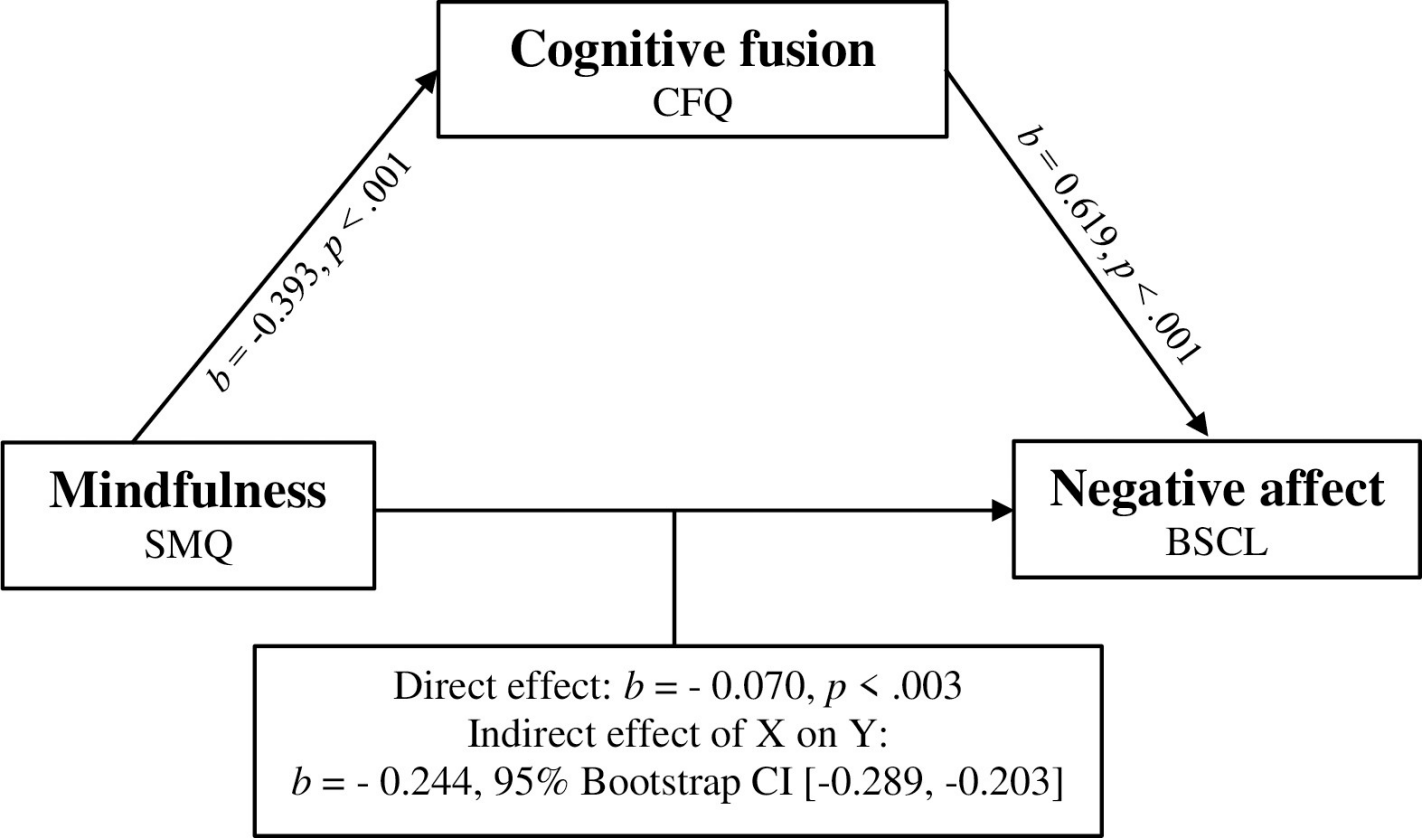
Model 4 Mediation model of mindfulness, cognitive fusion and negative affect. Model 4 mediation model of mindfulness, cognitive fusion and negative affect. SMQ: Southampton Mindfulness Questionnaire; CFQ: Cognitive fusion questionnaire; BSCL: Brief Symptom Checklist. Model controlled for age.

In the first step of examining the proposed moderated mediation model, the SPSS PROCESS macro Model 4 (see Fig 1) [54] was used to test the third hypothesis, namely whether cognitive fusion mediates the relation between mindfulness and negative affect. The overall model was statistically significant (*R*^2^ = .364, *F*(2, 736) = 197.60, *p* < .001). Furthermore, the relationship between mindfulness and the mediator cognitive fusion was significant (a: *b* = -0.393, *t*(736) = -30.238, *p* < .001) as well as the association between the mediator and negative affect (b: *b =* 0.619, *t*(735) = 11.72, *p* < .001). In addition, the direct relationship between mindfulness and negative affect without the mediator was significant (c: *b* = -0.314, *t*(736) = -16.713, *p* < .001). Given that the indirect effect (*b* = -0.248; 95% *CI* [-0.289, -0.203]) was found to be significant, with an effect size of R^2^ = 0.364, it can be concluded that there is a mediation effect. This is further supported by the finding that the relationship between mindfulness and negative affect weakened in magnitude once the mediator cognitive fusion was added (c’: *b* = -0.070, *t*(735) = -3.00, *p* = .003), suggesting that cognitive fusion mediates the relationship between mindfulness and negative affect. However, the finding that the direct effect between mindfulness and negative affect only decreased in significance after adding the mediator variable, instead of becoming nonsignificant, indicates that cognitive fusion is only a partial mediator of the association between mindfulness and negative affect. In partial mediation, the mediator is one variable that explains the relationship while not excluding the possibility that complex relationships are explained by more than one mediator. As complete mediation, which assumes the relationship is completely explained by a singular mediating variable is thought to be rare in the field of psychology [55] this finding is in line with our hypothesis.

**Fig 2:**
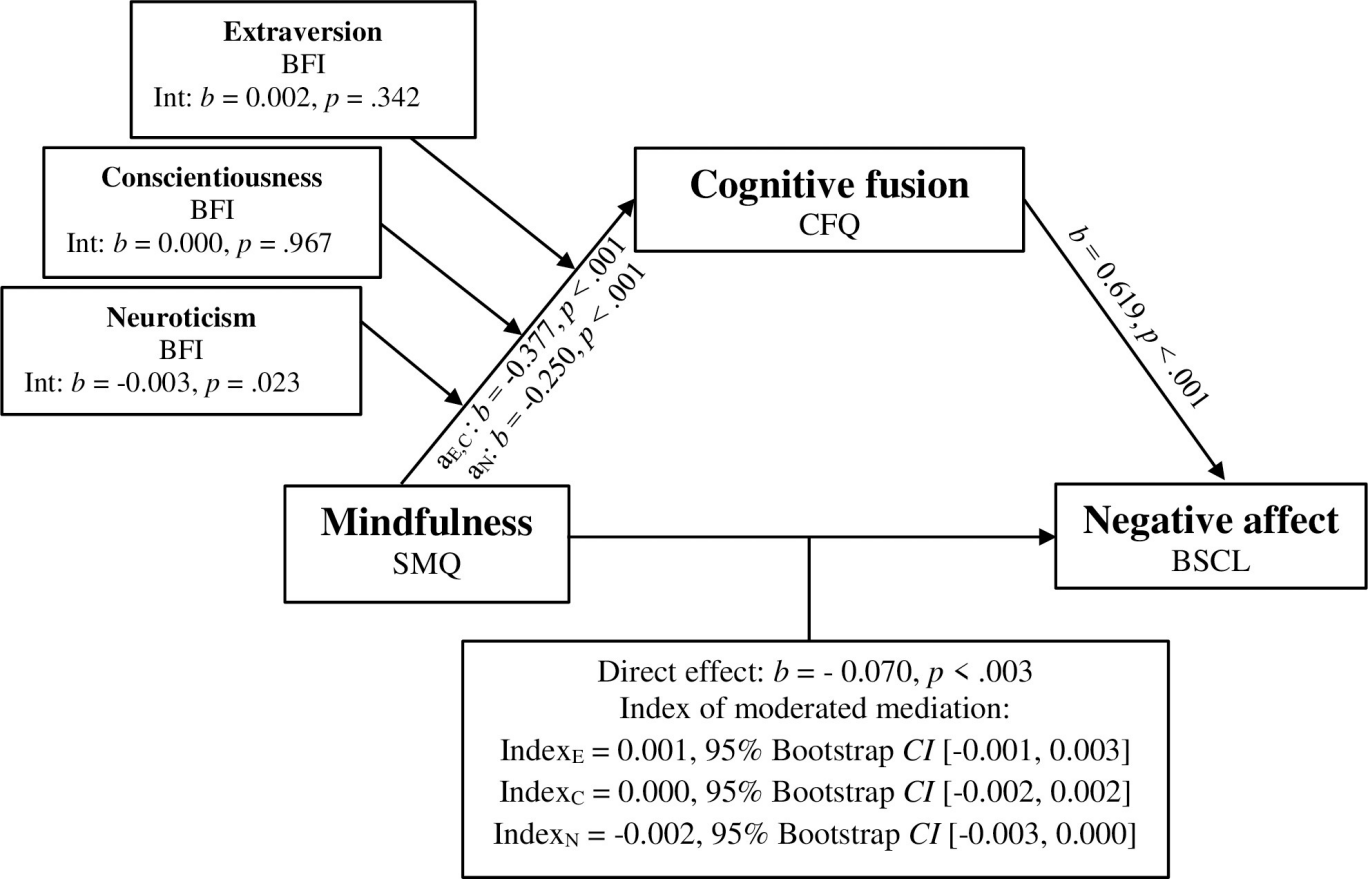
Model 7 moderated mediation model of mindfulness, cognitive fusion and negative affect with moderators extraversion, conscientiousness, and neuroticism. Model 7 moderated mediation model of mindfulness, cognitive fusion and negative affect with moderators extraversion, conscientiousness, and neuroticism. SMQ: Southampton Mindfulness Questionnaire, CFQ: Cognitive fusion questionnaire, BSCL: Brief Symptom Checklist, BFI: Big Five Inventory. Model controlled for age. Indexes of mediation refer to path-a.

Finally, to test the entirety of the proposed model, combining both the moderation and mediation components, three separate moderated mediation analyses were conducted using Model 7 of the PROCESS macro (see Fig 2) [54] with the moderators Extraversion (E), Conscientiousness (C), and Neuroticism (N). Parameters of the mediation component of the model remained significant with no notable changes from Model 4 (see Fig 2). It was hypothesized that the personality factors E, C, and N would moderate the relationship between mindfulness and cognitive fusion, thereby influencing the indirect relationship between mindfulness and negative affect. Contrary to our hypothesis, interaction effects between Mindfulness and the moderators E and C were not statistically significant (E: *b* = 0.002, *t*(734) = 0.951, *p* = .342; C: *b* = 0.000, *t*(734) = -0.041, *p* = .967). Correspondingly, the indexes of moderated mediation for E and C indicated that the moderators did not significantly affect the relationship between mindfulness and the mediator cognitive fusion (Index_E_: 0.001, 95% *CI* [- 0.001, 0.003], Index_C_: 0.000, 95% *CI* [- 0.002, 0.002]). As expanded on in Hayes [56], the index of moderated mediation refers to a formal test evaluating the linear association between the indirect effect and the assumed moderator of that effect. Though the interaction effect between Mindfulness and the moderator N was statistically significant (*b* = -0.003, *t*(734) = -2.271, *p* = .023), the confidence interval indicated by index of moderated mediation still included 0 (Index_N_: -0.002, 95% *CI* [-0.003, 0.000]). Given that the confidence interval touches 0, regardless of the significance of the t-test, there is insufficient evidence to conclude that the effect is significant.

## Exploratory analysis

In order to include a more comprehensive picture, including all traits of the five factor personality model, exploratory post-hoc analyses were performed testing the personality traits agreeableness (A) and openness (O). Exploratory tests investigating the two personality traits not included in the main model also did not show moderation effects for the personality factors agreeableness and openness. Interaction effects between Mindfulness and the moderators A and O were not statistically significant (A: *b* = 0.003, *t* (734) = 1.381, *p* = 0.168; O: *b* = -0.002, *t* (734) = -1.036, *p* = 0.301), in correspondence with the indexes of moderated mediation (A: *b* = 0.0019, 95% *CI* [-0.003, 0.004]; O: *b* = -0.0014, *CI* [0.0039, 0.001]).

## Discussion

The first objective of the present study was to investigate the role of cognitive fusion as a mediator, a central part of ACT and psychological flexibility, in the frequently reported relationship between mindfulness and negative affect in a diverse sample of frequent meditators from the general population. Secondly, we aimed to examine whether individual differences in terms of personality factors would moderate this model.

In line with various accounts in the literature [13, 57], the first hypothesis regarding the negative association between mindfulness and negative affect was supported by our data. Results showed a positive relationship between cognitive fusion and negative affect, as hypothesized and frequently evidenced in the literature [22, 58, 59].

To examine the role cognitive fusion may play with regard to positive effects of mindfulness, it was hypothesized that cognitive fusion mediates the negative association between mindfulness and negative affect. Our results support this theory in a population of frequent meditators, showing a partial mediation effect of cognitive fusion in the relationship of mindfulness and negative affect. In addition, the magnitude of this effect, as indicated by the large proportion of variance explained, further underlines this finding. Consonant with our findings, correlational evidence of this relationship in children was reported by [31]. Furthermore [60], showed longitudinal results of this mediating function of cognitive fusion in a clinical population. In conjunction with this evidence, our results strongly suggest that cognitive fusion, and analogously defusion, play an important role with regard to negative affect and attempts to alleviate it by means of mindfulness.

While our results did show a mediation effect of cognitive fusion, the finding that the direct effect between mindfulness and negative affect remained significant when the mediator was added to the model, indicates that the relationship is not fully mediated by cognitive fusion, indicating that additional parameters influence this mediation relationship. Possible additional mechanisms include experiential avoidance [21] and cognitive suppression, as suggested by [60].

Importantly, this evidence supports the assertion of mindfulness-based interventions, such as ACT, and that mindfulness has potential to reduce negative affect through cognitive fusion. Additionally, the results suggest that capacities trained by mindfulness and meditation (i.e., acceptance, non-judgment, non-reacting; [5]) likely counteract the maladaptive tendencies through which cognitive fusion is intricately linked with negative affect. In line with this assertion, as shown by [61, 62], psychologically inflexible individuals are less likely to employ contextually appropriate and adaptive emotion regulation strategies. Though it requires further investigation, our results suggest that possessing acquired mindfulness skills and automatically applying them as a way of defused thinking in daily life could prove an essential positive increment of emotion regulation strategies in making them more adaptive.

As described by [63], establishing boundary conditions of effects elucidates parameters for which an effect exists or does not exist, which has both theoretical and practical implications. Contrary to the suggested influence of personality characteristics on the effects of meditation and their relation to psychological flexibility [32, 35, 36], our hypothesis of personality as a moderator was not supported. Based on our data, the personality factors Extraversion, Conscientiousness, and Neuroticism do not appear to be such a boundary condition in a population of meditators. Hence, it appears that individuals may develop psychological flexibility through the practice of mindfulness regardless of specific underlying personality traits. It is not readily apparent from the available literature on mindfulness and personality as to why there was no significant moderating interaction effect. One possibility could be that this effect exists less in experienced meditators but plays a larger role in the general population and more novice meditators. When compared, scores on the Extraversion and Conscientiousness scales were consistent with norms values derived from an American general population sample [64]. Interestingly however, mean scores on the Neuroticism scale in this sample were lower than norm values [64], suggesting that this personality characteristic is less pronounced in frequent meditators. In addition, contrary to the possibility that meditators as a group may a priori share specific personality characteristics, making the group too homogenous to show moderator effects, evidence of insufficient variance was not evident in the sample. Secondly, given the correlational nature of the present study, and thus the proposed model, personality characteristics may have moderating effects at other locations in the model. This could include, for instance, at the direct effect between mindfulness and negative affect or between cognitive fusion and negative affect.

This being said, it is worth mentioning that Neuroticism showed the most potential as a moderator between mindfulness and cognitive fusion. This observation corresponds to Neuroticism consistently being found to be most strongly associated with mindfulness, psychological flexibility, and negative affect [32, 33, 36] and sharing considerable conceptual overlap with inflexibility and negative affect [65]. Importantly however, the found lack of moderation by personality characteristics affirms existing evidence of the transdiagnostic effects of mindfulness [40, 66]. In other words, this suggests that rather than there being a specific type of individual in which mindfulness may exert its benefits or personality factors that could limit the benefits of mindfulness on psychological flexibility, the effects of mindfulness are applicable across populations and personality types.

Lastly, the findings resulting from exploratory post-hoc analyses further cautiously point to a lack of personality as a boundary condition for the effects of mindfulness benefits on psychological flexibility and negative affect.

### Limitations

We acknowledge that the conclusions that can be drawn from this study are limited by several factors. Inherent to correlational data, such as ours, no causal inferences can be drawn. As such, as recommended by Winer et al. [38], the mediation model discussed in the present study is atemporal in nature. By extension, inferences regarding the definite positions of the examined variables in the model are, up until further investigation, interchangeable as no temporal precedence was assessed. Nonetheless, at initial stages of mediation analyses, correlational data still serve a worthwhile purpose by establishing mechanisms that show promise for further investigation [15, 63].

Secondly, as cognitive fusion was found to only be a partial mediator in the model, our results are limited in that only cognitive fusion was measured as a singular mediator. Further studies are needed to discern not only other factors explaining the relationship between mindfulness and negative affect but also the extent of the impact cognitive fusion has in relation to other mediators in the model. Considering that cognitive fusion is only one component of the broader concept of psychological flexibility, it may be that the construct of psychological flexibility as a whole could illuminate more about this relationship.

Furthermore, the present analysis included a largely Western sample. While it was attempted to address this issue during recruitment, this was in itself limited by the study only being available in English and outreach depending on meditation platforms, groups, and teachers that are represented on the internet. In addition, culturally grounded non-response bias, as discussed by [67, 68], can be assumed to have played a role in willingness to participate as a whole. While this is not avoidable in international cross-sectional studies, it does limit generalizability of results.

### Future directions

Despite the plethora of studies reporting beneficial effects, longitudinal studies are still scarce and, in some cases, have even reported lack of longitudinal effects and cumulative changes from mindfulness practice [13, 69]. The results shown in the present study point to an important relationship between mindfulness, psychological flexibility, and negative affect, as shown in correlational nature in a large and diverse sample. Thus, investigating this relationship in designs that allow for conclusions to be drawn is a necessary next step for future research. In particular, further longitudinal investigations are integral in establishing causality inferences, and could build upon the model suggested in the present study.

Given the partial mediation of the relationship between mindfulness and negative affect, experiential avoidance is a possible other mediator not examined in this study. Including this mediator in the model could elucidate more clearly the effect both cognitive fusion in comparison to experiential avoidance and psychological flexibility as a whole have in this relationship and essentially the mechanisms of positive effects of mindfulness. Moreover, as this relationship is clearly not explained by a single process, alternatives to traditional mediation analyses, such as dynamic network approaches, as suggested by [70], could prove useful in discerning the processes of change influencing the association between mindfulness and negative affect.

Finally, investigating the role personality factors may play at locations of the model that were not addressed in this study poses an interesting possibility for further research. Further exploration could elucidate the role personality plays as a boundary condition for the effects of mindfulness benefits on psychological flexibility and negative affect more clearly.

## Conclusion

The present study aimed to test a theory driven model of how mindfulness and personality characteristics are related to negative affect in frequent meditators. Our results add correlational data to support the mediating role of cognitive fusion. Limitations notwithstanding, the evidence from the current study goes beyond affirming cognitive fusion as a key parameter in this relationship. Namely, the mediation effect of cognitive fusion emphasizes that mindfulness should not be considered in isolation without regard to the relationship individuals have with their thoughts. Moreover, evidence from this population shows that meditation practice beyond and outside of mindfulness-based interventions can contribute to positive psychological health outcomes through the cultivation of adaptive, defused emotion regulation habits, and is not limited by personality characteristics.

## Supporting information

S1 Table Overview of meditation types in detail. N = 739. ^b^ Participants could choose multiple options; the total percentage may exceed 100%.
(DOCX)

S1 Dataset (SAV)
