# Supplementary material for: Cognitive fusion and personality traits in the context of mindfulness: A cross-sectional study
Source: PLoS One. 2022 Sep 28;17(9):e0273331. doi: 10.1371/journal.pone.0273331 (PMC9518896; doi:10.1371/journal.pone.0273331)
Supplement: S1 Table — N = 739. b Participants could choose multiple options; the total percentage may exceed 100%. (DOCX) [file pone.0273331.s001.docx]

**S1 Table.** **Overview of meditation types in detail**

| **Variable** | **Summary statistic – n (%)** | |
| --- | --- | --- |
| Meditation types^b^ |  |  |
|  | MBSR | 446 (56.5 %) |
|  | ACT | 78 (9.9 %) |
|  | Breath counting practice | 551 (69.8 %) |
|  | Jhana practice | 77 (9.8 %) |
|  | Body awareness practice | 530 (62.7 %) |
|  | Shamatha practice | 108 (13.7 %) |
|  | Mantra recitation | 310 (39.3 %) |
|  | Visualization | 412 (52.2 %) |
|  | Kirtan Kriya | 62 (7.9 %) |
|  | Cultivation of attention | 257 (32.6 %) |
|  | Choiceless awareness | 169 (21.4 %) |
|  | Vipassana / Insight | 234 (29.7 %) |
|  | Open monitoring meditation | 89 (11.3 %) |
|  | Analytical meditation | 51 (6.5 %) |
|  | Koan practice | 33 (4.2 %) |
|  | Four foundations of mindfulness | 107 (13.6 %) |
|  | Mahamudra | 53 (6.7 %) |
|  | Dzogchen | 37 (4.7 %) |
|  | Muragaba | 4 (0.5 %) |
|  | Shikantza | 116 (14.7 %) |
|  | Self-inquiry | 166 (21.0 %) |
|  | Metta meditation | 377 (47.8 %) |
|  | Compassion cultivation training | 128 (16.2 %) |
|  | Bodhichitta | 85 (10.8 %) |
|  | Centering prayer | 64 (8.1 %) |
|  | Cognitive-based compassion training | 40 (5.1 %) |
|  | The six recollections | 12 (1.5 %) |
|  | Contemplation of mortality | 66 (8.4 %) |
|  | Wellbeing therapy | 63 (8.0 %) |
|  | Development stage | 18 (2.3 %) |
|  | Meditation on foulness | 18 (2.3 %) |
|  | Yoga | 543 (68.8 %) |
|  | Other | 105 (13.3 %) |

N = 739.

^b^ Participants could choose multiple options; the total percentage may exceed 100%.
